# Does sports participation reduce disparities in anxiety and depression symptoms among sexual minority and heterosexual undergraduate students? A cross-sectional study

**DOI:** 10.3389/fpubh.2026.1838233

**Published:** 2026-04-30

**Authors:** André Felipe Oliveira da Cruz, Pedro Victor Felisberto da Silva, Mateus Sanches Jokura, Jessenia Marise Sales Campos, Marcelo Augusto Alves dos Santos, Thaís Aparecida Medeiros Amorim, Giovanni Henrique Quizzini, Matheus Augusto Martins Correia, Ana Paula Rodrigues Rocha, Camila Buonani, Sueyla Ferreira da Silva dos Santos, Andrea Deslandes, Lucas Melo Neves, Fabricio E. Rossi

**Affiliations:** 1 Sports and Strength Exercise Research Group, Department of Physical Education, Faculty of Science and Technology, São Paulo State University (UNESP), São Paulo, Brazil; 2 Graduate Program in Movement Science - Interunits, São Paulo State University (UNESP), São Paulo, Brazil; 3 Unidade de Pesquisa Clínica, National Institute of Women, Children and Adolescents Health Fernandes Figueira (IFF), Oswaldo Cruz Foundation (FIOCRUZ), Rio de Janeiro, Brazil; 4 Department of Physical Education, Faculty of Science and Technology, São Paulo State University (UNESP), São Paulo, Brazil; 5 Health and Movement Research Group, Department of Physical Education, Faculty of Science and Technology, São Paulo State University (UNESP), São Paulo, Brazil; 6 Institute of Psychiatry, Federal University of Rio de Janeiro, Rio de Janeiro, Brazil; 7 Laboratory of Physical Activity, Sport, and Mental Health (LAFESAM), São Paulo State University (UNESP), Institute of Bioscience, Rio Claro, Brazil; 8 Bipolar Disorder Program (PROMAN), Department of Psychiatry, University of São Paulo Medical School, São Paulo, Brazil; 9 Graduate Program Human Development and Technologies, São Paulo State University (UNESP), Rio Claro, Brazil

**Keywords:** college students, minority stress, sexual orientation, sports participation, student-athletes

## Abstract

**Introduction:**

Although sports participation is recognized for its psychological benefits, it remains unclear whether participation in competitive sports may produce different effects on these symptoms among sexual minority university students compared to their heterosexual peers. This study aimed to compare anxiety and depression symptoms between 524 undergraduate students engaged and not engaged in sports competitions by sexual orientation.

**Methods:**

With cross-sectional design, this study included 524 undergraduate students, categorized as engaged in sports participation or not engaged, and by sexual orientation (bisexual, gay/lesbian, and pansexual). Anxiety and depression symptoms were assessed using the Generalized Anxiety Disorder-7 and Patient Health Questionnaire-9.

**Results:**

Sexual minority students (bisexual, gay/lesbian, and pansexual) with sports participation had a significantly higher prevalence of moderate to severe anxiety (50.5% vs. 30.2%) and depression symptoms (50.5% vs. 35.6%), as a higher risk for anxiety (OR = 2.4 95% CI: 1.38 to 4.03, *p* = 0.002) and depression symptoms (OR = 1.8 95% CI: 1.09 to 3.13, *p* = 0.022) compared to heterosexual students with sports participation. Gay/lesbian students with sports participation had significantly higher anxiety (11.4 ± 5.4 vs. 7.8 ± 4.7, *p* = 0.007) and depression scores (13.2 ± 7.0 vs. 8.7 ± 5.9, *p* = 0.006) than heterosexual students with sports participation. However, there was no significant difference in the students without sports participation.

**Discussion:**

Sports participation did not appear to minimize anxiety or depression symptoms among sexual minority students, particularly among gay/lesbian, highlighting the need for more inclusive and supportive university sports environments.

## 1 Introduction

University life represents a period of significant challenges and transitions, filled with stressful factors such as excessive study load, pressure for academic performance, and dealing with new responsibilities (1). It involves a series of social interactions that demand academic and emotional competencies, which can harm mental health or precipitate the emergence of elevated symptoms of anxiety and depression. When left untreated, these can progress into mental disorders (2, 3).

Leão et al. (4) found that approximately 36.1% of university students exhibit higher levels of anxiety, while 28.6% display symptoms of depression. Similarly, Lima et al. (5) reported a higher incidence of moderate to severe anxiety symptoms at 42.5% and depression symptoms at 51.0% among university students (5). The incidence of such symptoms has a significant impact on academic performance, with deleterious effects on productivity (6), and may contribute to an increase in university dropout rates (7). The importance of mental health among the university population is crucial, and we highlight it specifically in relation to sexual orientation (heterosexual, gay/lesbian, bisexual, asexual, and pansexual).

Sexual orientation is a multidimensional construct and a combination of attraction, identity, interests, and behavior (8). Incidences of adverse mental health outcomes (e.g., depression, anxiety) are elevated among bisexual, gay/lesbian, pansexual, and asexual individuals in comparison to heterosexual individuals (9). This elevated prevalence is explained by the exposure of sexual minority individuals to unique, chronic stressors associated with their stigmatized social status, including discrimination, prejudice, and internalized stigma (10, 11).

In fact, the meta-analysis conducted by Lucassen and collaborators indicated that the proportion of sexual minority youth (e.g., gay, lesbian, and bisexual individuals) ranges from 2.3 to 12% of university students, with sexual minority individuals exhibiting elevated odds rates of depressive symptoms and depressive disorder (odds ratio = 2.94, *p* < 0.001, and standardized mean difference, d = 0.39, *p* < 0.001) relative to heterosexual peers (9). In other words, sexual minority youth face the double stigma of belonging to a marginalized group while also having mental health issues.

A widely recognized strategy for its benefits is the regular practice of physical activity for mental health (12–14). Encouraging regular physical activity among university students, such as sports, walking, or gym workouts, proves effective in improving psychological well-being, especially when compared to sedentary students (15). Furthermore, sports participation contributes to increased self-esteem, improved cognitive function, and engagement in support networks, promoting a sense of community and social interaction, which are essential factors for mental health during the transition to university life (15).

The study by Souza et al. (16) with university students found that medical students engage in less moderate and vigorous physical activity and present more anxiety symptoms compared to Physical Education students. These results reinforce that physical inactivity is associated with higher levels of anxiety among medical students, while the more active Physical Education students report fewer anxiety symptoms. In the sports context, Kader et al. (17) found a 40% lower risk of a diagnosis of depression and anxiety disorders in elite soccer players from the Swedish first division compared to the general male population, reinforcing the importance of sports practice and its positive implications for mental health.

Despite the importance of physical activity and sports participation for improving mental health (18), it is still unclear whether university students belonging to sexual minority populations, in comparison to heterosexual students, with sports participation present differences in anxiety and depression symptoms. It is hypothesized that sexual minority students with sports participation may have a protective factor, minimizing the risk of elevated anxiety and depression symptoms. Therefore, the aim of this study was to compare anxiety and depression symptoms between undergraduate students engaged and not engaged in sports participation, considering sexual orientation.

## 2 Materials and methods

### 2.1 Sample

The study was approved by the Research Ethics Committee of São Paulo State University “Júlio de Mesquita Filho” (CAAE: 69741023.0.0000.0081; Protocol No.: 6.131.384). All volunteers signed the Free and Informed Consent Form. The inclusion criteria were: (1) being an undergraduate student regularly enrolled at São Paulo State University.

The UNESP comprises 34 university units distributed across 24 municipalities in the state of São Paulo, totaling 35,791 undergraduate students as of March 2025. The sample size calculation was based on data from a previous study conducted by Sheldon et al. (19), which identified a prevalence of 25% of depressive symptoms among undergraduate students. Following the methodological recommendations proposed by Miot (20), a tolerable margin of error of 4% and a significance level of 5% (Z = 1.96) were adopted, resulting in a simple random sample of 445 participants.

Participants were classified into four categories according to sexual orientation: heterosexual or sexual minority (minority group included individuals who self-identified as bisexual, gay/lesbian, pansexual, or asexual) (21–23); and sports participation: engaged or not engaged in sports participation.

Undergraduate students with at least 1 year of participation in college sports competitions were classified as engaged in sports participation. On average, they trained three times per week for approximately 90–100 min per session. These students competed in two major recreational events during the year, the UNESP Cup and the College Games, which involve students from different campuses and various sport disciplines, including futsal, soccer, indoor volleyball, beach volleyball, basketball, handball, swimming, track and field, judo, table tennis, tennis, and chess. The other group was not engaged in college sports competitions, although they could be physically active.

### 2.2 Procedures

The University currently offers 136 undergraduate courses, distributed across 186 admission modalities. These courses cover a total of 64 majors, with 17 in the Biological Sciences area, 24 in the Exact Sciences, and 23 in the Humanities.

Data collection instruments were applied using Google Forms®, a digital platform also accessible on mobile devices. The participation link was made available through the university’s institutional system and disseminated on social media. Participants were recruited through convenience sampling using institutional dissemination channels and social media platforms.

### 2.3 Measures

#### 2.3.1 Anxiety symptoms

Anxiety symptoms were measured using the Generalized Anxiety Disorder-7 (GAD-7) instrument, a self-reported questionnaire consisting of seven items, developed to assess an individual’s anxiety state in the 2 weeks prior to administration (24). Participants were instructed to assign a score from 0 to 3 for each item, according to the frequency with which the symptoms bothered them in the last 2 weeks (0 = “never,” 1 = “several days,” 2 = “more than half the days”, 3 = “nearly every day”). Using a cut-off point of 10, the GAD-7 has a sensitivity of 89% and a specificity of 82% for diagnosing generalized anxiety disorder (25).

#### 2.3.2 Depression symptoms

Depressive symptoms were assessed using the Patient Health Questionnaire-9 (PHQ-9) (26) validated for the Portuguese language (27). Items are scored from 0 to 3 using the same response options described for the GAD-7. The scores range from 0 to 27, a total score equal to or greater than 10 is recommended as the cut-off point for screening for a major depressive episode, presenting a sensitivity and specificity of 88% each (26).

#### 2.3.3 Sexual orientation

Sexual orientation was measured according to the classification established by the Coordination of Policies for Sexual Diversity (5th ed.), which categorizes it as heterosexual, bisexual, gay/lesbian, pansexual, and asexual (28).

#### 2.3.4 Social determinants

Biological sex and race were assessed through self-report. Participants were asked to indicate their biological sex by selecting one of the following options: male or female. Race was self-reported according to the alternatives: White, Black, Asian, multiracial, and Indigenous.

### 2.4 Statistical analysis

Descriptive analysis was performed for sample characterization, and the Student’s t test for independent samples was used to verify differences between student engaged or not in sports participation. The Levene test was used to verify the homogeneity of variance of the data set, in addition to one-way analysis of variance (ANOVA), followed by Tukey’s *post-hoc* test, to verify possible differences within groups.

For categorical outcomes, the chi-square test (with Yates’ correction used in two-by-two contingency tables) was applied to assess the existence of associations between dependent and independent variables (crude analysis). The bivariate logistic regression model was adopted and was expressed as odds ratios (OR) and their 95% confidence intervals (95% CI). The goodness-of-fit assumption was assessed using the Hosmer–Lemeshow test. Statistical significance was set at *p* < 0.05. The data were analyzed using the Statistical Package for the Social Sciences, version 29.0 (SPSS Inc., Chicago, IL, United States).

Due to the very small number of participants who identified as asexual (*n* = 2), this group was excluded from the statistical analyses, as the sample size was insufficient to allow meaningful statistical comparisons.

## 3 Results

The descriptive characteristics of the sample are summarized in Table 1. The students engaged in sports (*n* = 244) and students not engaged in sports (*n* = 280) were comparable in age, with mean values of 21.8 ± 3.8 and 22.6 ± 5.7 years, respectively. Body weight and height were also similar between groups, although students engaged in sports exhibited a slightly higher mean height (171.7 ± 10.4 cm) compared to students not engaged in sports (169.6 ± 9.6 cm). As expected, those who practiced sports reported substantial training experience, averaging 5.6 ± 4.7 years of practice, alongside a weekly training frequency of 3.0 ± 1.4 sessions and approximately 106.0 ± 29.8 min of training per day; these variables were not applicable to the not engaged in sports participation group.

**Table 1:** Characteristics of the sample.

Categorical variables	Variables	Students engaged in sports participation (*n* = 244)	Students not engaged in sports participation (*n* = 280)
		Mean (SD)	Mean (SD)
	Age (year)	21.8 ± 3.8	22.6 ± 5.7
	Weight (kg)	71.8 ± 16.9	71.0 ± 15.2
	Height (cm)	171.7 ± 10.4	169.6 ± 9.6
	Experience (year)	5.6 ± 4.7	-
	Frequency per week (time)	3.0 ± 1.4	-
	Time per day (min)	106.0 ± 29.8	-
		N (%)	N (%)
Race	White	157 (64.3)	184 (65.9)
	Black	24 (9.8)	26 (9.3)
	Asian	11 (4.5)	13 (4.7)
	Indigenous	-	1 (0.4)
	Multiracial	52 (21.3)	55 (19.7)
Sexual orientation	Women	129 (57.6)	140 (61.1)
	Heterosexual	149 (61.1)	191 (68.0)
	Bisexual	63 (25.8)	58 (20.6)
	Gay/lesbian	22 (9.0)	18 (6.4)
	Pansexual	8 (3.3)	11 (3.9)
	Asexual	2 (0.8)	2 (0.7)

SD, standard deviation.

Regarding sexual orientation, heterosexual students represented 60.6% of engaged and 68.0% of not engaged in sports participation. Sexual minority students accounted for 38.6% of the engaged in sports participation sample and 31.7% of the not engaged in sports participation group. Bisexual individuals comprised the largest subgroup within the sexual minority group in both students engaged (25.6%) and not engaged in sports (20.6%). Gay participants represented 8.9% of students engaged and 6.4% of not engaged in sports, while pansexual individuals accounted for 3.3 and 3.9%, respectively. Asexual individuals were the smallest subgroup, with 0.8% among students engaged and 0.7% among not engaged in sports, and were not included in the main analysis.

**Figure 1:**
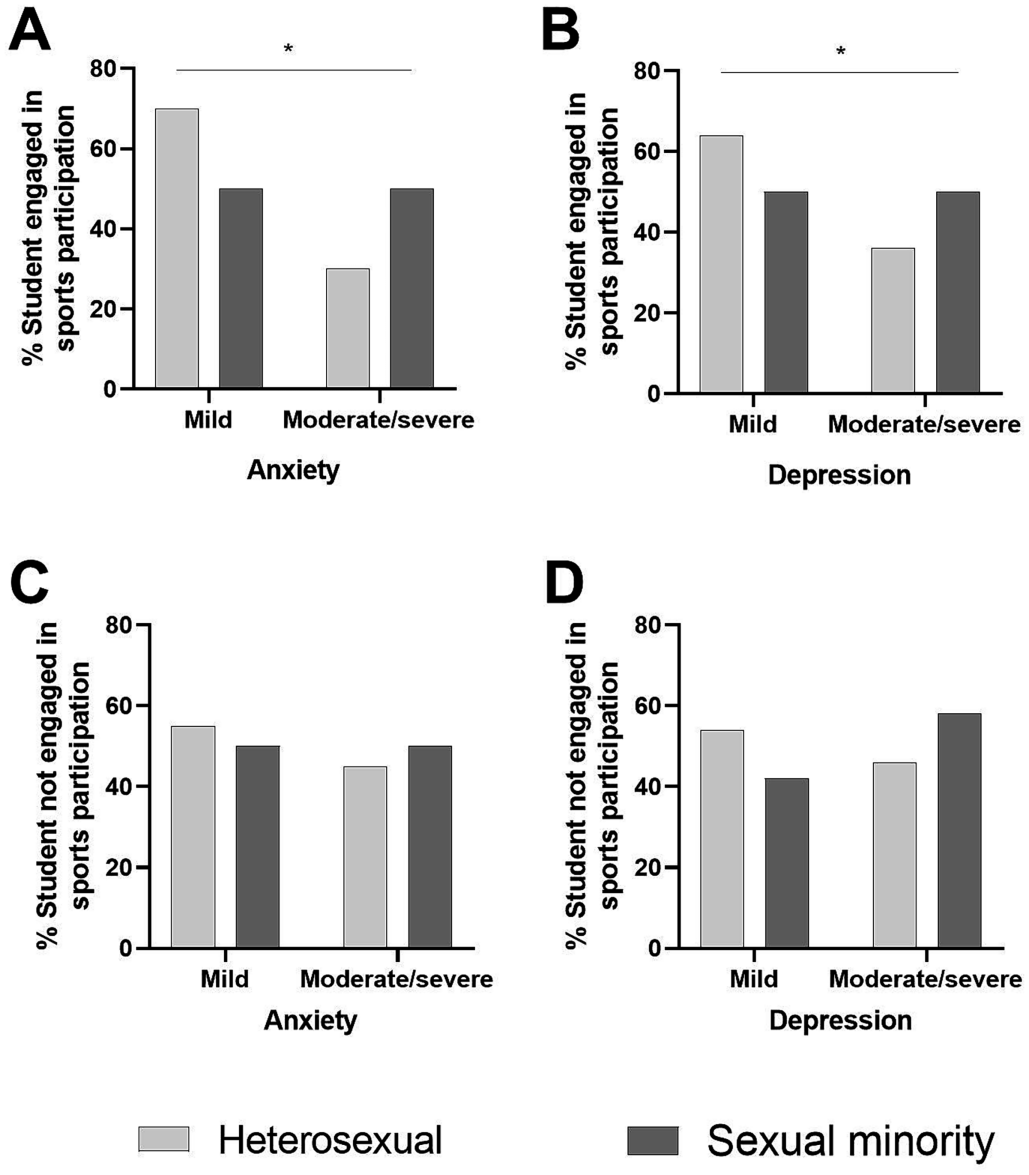
Comparison of prevalences of anxiety and depression symptoms according to heterosexual or sexual minority status among students engaged or not engaged in sports participation. Panel **(A)** – Students engaged in sports participation and anxiety prevalence; Panel **(B)** – Students engaged in sports participation and depression prevalence; Panel **(C)** – Students not engaged in sports participation and anxiety prevalence; Panel **(D)** – Students not engaged in sports participation and depression prevalence. Symptom severity: 0–9 = mild; ≥10 = moderate/severe. Sexual minority: bisexual, gay, and pansexual. Chi-square test, *p < 0.05.*

Figure 1 presents the comparison of prevalences between sexual minority students and heterosexual students and the severity of anxiety and depression symptoms among students engaged or not in sports participation, as determined by the chi-square test.

Among students engaged in sports participation, the comparison of prevalence between heterosexual and sexual minority groups showed significant differences for both anxiety (*p* = 0.002) and depression symptom severity (*p* = 0.030). A higher proportion of sexual minority students engaged in sports presented moderate to severe anxiety symptoms (50.5%) compared with heterosexual students engaged in sports (30.2%). Similarly, the prevalence of moderate to severe depression symptoms was higher among sexual minority students engaged in sports (50.5%) than among heterosexual counterparts (35.6%).

Among students not in sports participation, the comparison of prevalence between heterosexual and sexual minority groups revealed no significant differences in anxiety (*p* = 0.581) or depression symptom severity (*p* = 0.102). The proportion of moderate to severe symptoms was slightly higher among sexual minority students compared with their heterosexual counterparts, but the differences did not reach statistical significance.

**Figure 2:**
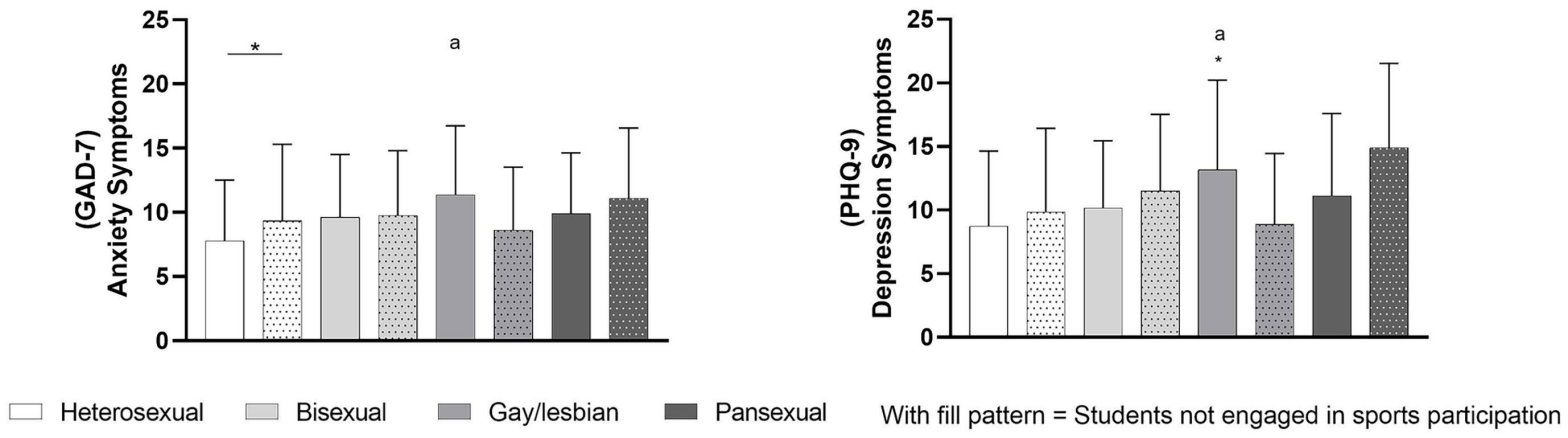
Comparison of anxiety and depression symptoms among students engaged or not in sports participation, stratified by sexual orientation. (a) Significant difference compared with heterosexual individuals; (*) significant difference between students engaged in sports participation and students not engaged in sports participation; GAD-7, Generalized Anxiety Disorder-7; PHQ-9, Patient Health Questionnaire-9.

Figure 2 presents the comparison of anxiety and depression symptoms among students engaged or not in sports participation, stratified by sexual orientation.

When comparing heterosexual students between students engaged or not in sports participation, those who were not engaged exhibited significantly higher anxiety symptoms than their counterparts who were engaged in sports (*p* = 0.009). Regarding sexual orientation within groups, one-way ANOVA revealed a significant effect of sexual orientation among students engaged in sports (*p* = 0.002), but not among who were not engaged in sports (*p* = 0.672). Tukey’s *post-hoc* analysis indicated that gay/lesbian students engaged in sports presented higher anxiety scores compared with heterosexual counterparts (*p* = 0.007).

For depression symptoms, between-group comparisons indicated that gay/lesbian who were not engaged in sports reported lower depression symptoms than gay/lesbian engaged in sports (*p* = 0.042), whereas no significant differences were found between groups in other sexual orientation categories. There were significant differences among students engaged in sports (*p* = 0.007) and those not engaged (*p* = 0.024). Gay/lesbian engaged in sports participation showed higher depression scores compared with heterosexual counterparts (*p* = 0.006), but Tukey’s *post hoc* analysis identified only a trend toward a significant difference between pansexual and heterosexual individuals in those students not engaged in sports participation (*p* = 0.056).

**Figure 3:**
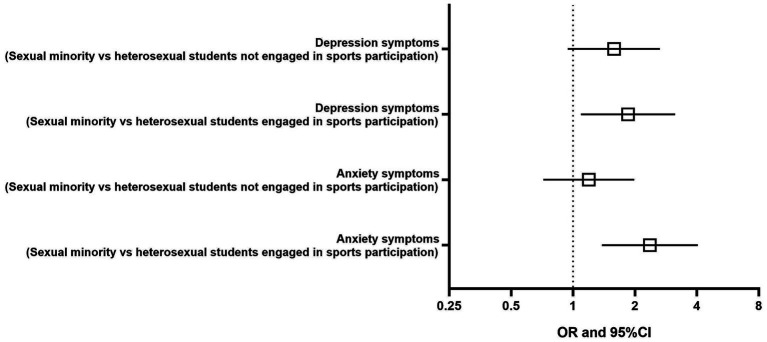
Binary logistic regression between sexual orientation (heterosexual vs. sexual minority) and the severity of anxiety and depression symptoms among students engaged or not in sports participation. OR, odds ratio; CI, confidence interval.

In the bivariate analysis (Figure 3), anxiety symptoms were significantly associated with sexual orientation only in the students engaged in sports participation (OR = 2.4 95% CI: 1.38 to 4.03, *p* = 0.002), but not in the students not engaged in sports (OR = 1.2 95% CI: 0.72 to 1.98, *p* = 0.495).

Depression symptoms were also significantly associated with sexual orientation in the students engaged in sports participation (OR = 1.8 95% CI: 1.09 to 3.13, *p* = 0.022), but not in the students not engaged in sports (OR = 1.6 95% CI: 0.94 to 2.63, *p* = 0.079).

When examining the odds ratios for each sexual minority group, stratified by sex, in relation to moderate to severe anxiety or depression symptoms, no significant associations were found (*p* > 0.05).

## 4 Discussion

The results obtained in this study showed that sexual minority undergraduate engaged in sports participation presented a higher prevalence of moderate to severe anxiety and depression symptoms compared to heterosexual counterparts. Additionally, we identified a complex and multifaceted scenario regarding the association between sexual orientation, mental health, and sports practice, with higher and significant anxiety and depression symptom scores in gay/lesbian engaged in sports participation compared to heterosexual students engaged in sports. However, this difference was present only in the student engaged in sports participation, suggesting the university sports environment as a potential amplifier of existing disparities in the mental health of this population.

Our results, showing lower anxiety symptom scores in heterosexual students engaged in sports compared to their counterparts not engaged in sports, are consistent with the study conducted by Edwards e Froehle (29), which compared college athletes and non-athletes while considering not only sports practice but also participation in organized sports or athletic activities at the university level, taking into account previous diagnoses of anxiety disorders. Although the American study did not analyze sexual orientation, its findings provide an important benchmark, suggesting that involvement in organized sports activities may be associated with reduced anxiety levels, an effect similar to that observed in our sample.

We expected that sexual minority individuals would present higher anxiety and depression symptoms and that this disparity would be attenuated in the group of students engaged in sports participation. Regarding the higher mean scores of gay university students compared to heterosexual students in anxiety and depression symptoms, our findings align with results presented in the literature, such as the study by Borgogna et al. (30), which also used the GAD-7 and PHQ-9 scales and found disparities between heterosexual and sexual minority individuals.

This hypothesis was not fully confirmed, as we observed this disparity only in the group of students engaged in sports participation, as well as when comparing depressive symptoms between gay/lesbian engaged and not engaged in sports, with those who were engaged in sports participation presenting higher scores. This leads to possible interpretations that the competitive university sports environment, while potentially a protective factor for heterosexual engaged in sports participation, may be associated with greater exposure to minority stressors among sexual minority students, reported by Meyer (10) for gay individuals. Minority stress theory describes the adversities in the social environment in which individuals are embedded; considering sexual orientation, these may include stigma, prejudice, discrimination, and expectations of rejection, which, when internalized, cause a worsening of mental health (10).

Other influencing factors can be explained, in part, by prior studies indicating the prevalent use of homophobic language in sport, which may adversely affect the mental health of sexual minority in sports participation (31). Denison et al. (31) indicated that more than half of the participants reported encountering homophobic language at least once in the preceding 2 weeks. Contributing to these findings, the American Medical Society for Sports Medicine links homophobic language to various adverse health outcomes for sexual minority individuals, including concerns regarding mental health (32).

In addition, Xiang et al. (33) describe the experiences of sexual minority student-athletes in university sports, reporting that such individuals experience constant discrimination and violence in this environment (abuse, harassment, threats), as well as stigma, stereotypes, and cultural or religious pressures, leading to internalization and even self-aversion. This would justify the findings in the students engaged in sports participation of the present study, although, when inserted into an environment with a support network, these individuals can develop greater resilience, becoming stronger and more confident.

Another noteworthy finding is that only gay/lesbian individuals presented elevated symptoms when compared to heterosexual individuals. According to various studies, such as the meta-analysis by Wittgens et al. (34), the opposite pattern has been reported, with bisexual individuals showing the greatest disparities, followed by gay/lesbian individuals. Our findings in this regard may have diverged due to differences in sample size, or they may demonstrate that, in our sample, disparities related to bisexuality were attenuated. However, future studies are needed to confirm this hypothesis.

However, it should be noted that this study also has certain limitations. First, the observational cross-sectional design hampers establishing cause-and-effect relationships. Second, as observed in the study by Wittgens et al. (34), disparities according to sexual orientation were greater in studies using more robust methods. In addition, although the study attempted to include sexual minority students, some sexual orientations, such as asexuality, were not considered in the comparisons due to insufficient sample size. Furthermore, the lack of control over contextual variables, such as psychosocial factors, history of discrimination, religiosity and identity, as well as socioeconomic status, academic year or semester, field of study, prior psychiatric diagnosis, social support, and characteristics of the sports environment (e.g., team climate or coach support), may have influenced the results. It is also possible that the cultural context of Brazilian universities, characterized by regional diversity and differences in the structure of university sports, impacted the perception and reporting of stress. Despite the previous limitations, this study contributes special insights into the context of sports participation, a topic of significant social importance and public health implications. Future studies should adopt longitudinal designs and use specific instruments to measure minority stress, as well as consider the inclusion of contextual variables in order to better understand the psychosocial mechanisms involved in the mental health of sexual minority students engaged in sports participation.

## 5 Conclusion

University sports participation did not appear to minimize anxiety or depression symptoms of sexual minority students. On the contrary, the competitive environment may be associated with greater exposure to minority stressors, especially for gay/lesbian engaged in sports participation, exacerbating mental health disparities. The results suggest the need to create more inclusive university sports environments and support networks through initiatives aimed at reducing discrimination, strengthening social support, and promoting awareness of sexual diversity, particularly within the university sports context.

## Data Availability

The raw data supporting the conclusions of this article will be made available by the authors, without undue reservation.
